# Efficacy of Endoscopic Retrograde Cholangiopancreatography and Frey Procedure in the Treatment of Pediatric Pancreatic Duct Stones: A Single-Center Retrospective Cohort Study

**DOI:** 10.3390/children12111555

**Published:** 2025-11-17

**Authors:** Zhenyu Xie, Yifan Deng, Chengkun Luo, Yun Peng, Yang Chen, Jiulin Song

**Affiliations:** Department of Pediatric Surgery, West China Hospital, Sichuan University, #37 Guo-Xue-Xiang, Chengdu 610041, China; zhenyuxie990201@gmail.com (Z.X.); 18dengyifan@sina.com (Y.D.); 13408099257@163.com (C.L.); pengyunjiayouya@163.com (Y.P.); yangchenwch@scu.edu.cn (Y.C.)

**Keywords:** children, chronic pancreatitis, pancreatic duct stones, endoscopic retrograde cholangiopancreatography, frey procedure

## Abstract

**Objective:** To compare the efficacy of endoscopic retrograde cholangiopancreatography (ERCP) and the Frey procedure in the treatment of pediatric pancreatic duct stones (PDS). **Methods:** A retrospective analysis was conducted on 65 pediatric patients treated for pancreatic duct stones in the Department of Pediatric Surgery, West China Hospital of Sichuan University, between February 2018 and May 2025. Demographic data, perioperative clinical parameters, postoperative recovery, and complications were collected. The efficacy and complications of ERCP and the Frey procedure were evaluated. **Results:** Of the 65 patients, 37 (56.92%) were male and 28 (43.08%) were female, with a median surgical age of 14 (11, 16) years. 32 patients (49.23%) underwent ERCP, and 33 patients (50.77%) underwent the Frey procedure. Significant differences were observed between the two groups in the degree of main pancreatic duct dilation (6.45 vs. 9.11, *p* < 0.001), postoperative stone recurrence (13 vs. 3, *p* = 0.003), and number of reinterventions (3.98 vs. 1.27, *p* < 0.001). The 5-year intervention-free survival rate was 57.75% in the ERCP group and 88.86% in the Frey group, with a statistically significant difference between groups (*p* = 0.024). **Conclusions:** Both ERCP and the Frey procedure are effective for pediatric PDS. ERCP is preferred for patients with mild ductal dilation and first-onset stones. However, for those with significant ductal dilation or recurrent stones with suboptimal ERCP outcomes, the Frey procedure is recommended.

## 1. Introduction

Pancreatic calculi are commonly regarded as a complication of chronic pancreatitis (CP) and are often identified during long-term follow-up of CP patients [1,2]. However, the etiology of pediatric pancreatic calculi differs significantly from that of adults. However, in pediatric pancreatic duct stones, the primary cause is pancreaticobiliary maljunction, leading to the normal secretory function of the pancreas being impaired. and the ensuing ductal and parenchymal hypertension often causes severe pain [3,4].

This study aims to compare the clinical outcomes, efficacy, and safety of ERCP versus Frey for the management of pediatric PDS.

## 2. Data and Methods

### 2.1. Clinical Data

A retrospective analysis was conducted on the clinical data of 65 pediatric patients with PDS who underwent ERCP or the Frey procedure at the Department of Pediatric Surgery, West China Hospital of Sichuan University, between February 2018 and May 2025. Among them, 32 patients received ERCP and 33 underwent the Frey procedure. Inclusion criteria were ① preoperative diagnosis of CP; ② imaging evidence of pancreatic duct stones with associated dilatation; ③ complete follow-up data. Exclusion criteria were ① incomplete clinical data and ② loss to follow-up.

This study was approved by the Ethics Committee of West China Hospital, Sichuan University (Approval No. 202435), and written informed consent was obtained from all patients’ guardians.

Collected clinical data included: ① Baseline characteristics: gender, age, height, weight, body mass index (BMI); ② Perioperative data: type of pancreatic duct stone (single or multiple), stone size, degree of main pancreatic duct dilatation (DMPDD); preoperative and postoperative laboratory indices including: white blood cell (WBC) count, C-reactive protein (CRP), amylase (AMY), lipase(LIP), alanine aminotransferase (ALT), total bilirubin (TB), fasting blood glucose (FBG), and albumin (ALB); ③ Prognostic indicators: pain relief (PR) (complete relief: Izbicki pain score ≤10; partial relief: Izbicki pain score >10 with >50% reduction), multiple interventions (MI) (ERCP ≥ 3 or Frey ≥ 2), and complications: postoperative pancreatitis (PP), postoperative bleeding (PB), pancreatic fistula (PF), intra-abdominal infection (IAI), stone recurrence (SR).

**Figure 1:**
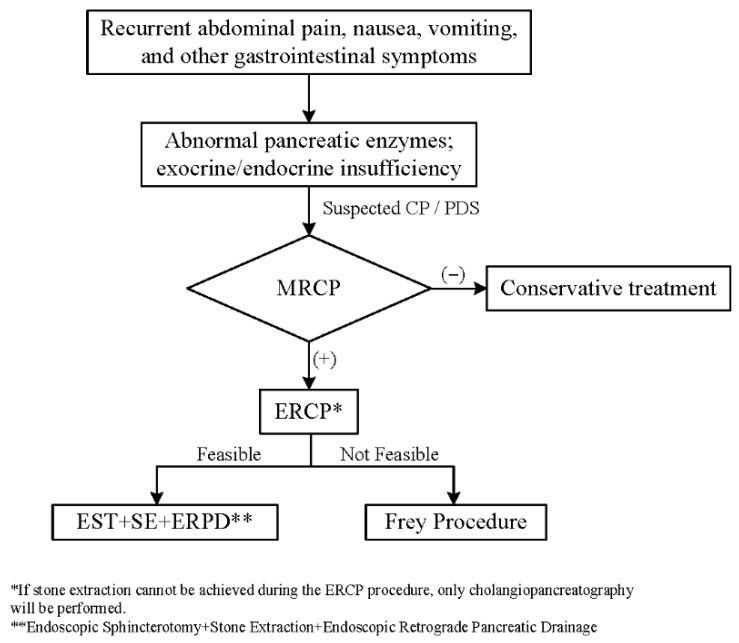
Patient care flowchart.

For the detailed diagnostic and treatment flowchart, please refer to the patient care flowchart (Figure 1).

### 2.2. Surgical Approaches

ERCP: the patient is placed in the prone position, and a duodenoscope is advanced into the descending duodenum. After successful pancreatic duct cannulation, 5 mL of 25% Omnipaque (Cork, Ireland) is injected using an arched incision knife. Under fluoroscopy, pancreatic duct stricture and stones are confirmed. A pull-type sphincterotome is inserted, and electrosurgical currents are applied to incise the papillary sphincter. The pancreatic duct is dilated with an appropriately sized catheter, and a guidewire-assisted stone retrieval basket is used to extract white stones, which are discharged into the intestine. A plastic stent of suitable size is placed along the guidewire into the main pancreatic duct for drainage and secured in place. After confirming smooth pancreatic juice drainage, the procedure is completed, and the patient is advised to undergo stent replacement or removal in 3–6 months. After the procedure, the biliary tract is irrigated, followed by cholangiopancreatography to confirm that no residual stones remained. Three days postoperatively, the patient’s symptoms and laboratory findings improve significantly. Before discharge, a follow-up MRCP is performed, and the absence of residual stones indicated that the stones have been completely removed.

Frey: a transverse upper abdominal incision was made to enter the abdominal cavity. The duodenum was mobilized using the Kocher maneuver to expose the pancreatic head, and the lesser sac was opened by dividing the gastrocolic ligament. The main pancreatic duct was identified, longitudinally opened from the tail to the head, and explored to remove intraductal calculi. The patency of the main pancreatic duct and the papilla was confirmed by gentle probing. A limited resection of the pancreatic head was then performed. Fibrotic and inflammatory tissue within the pancreatic head was cored out along the main duct and its branches, while preserving a thin rim of viable parenchyma and the posterior capsule to protect the common bile duct and duodenum. This decompressed the pancreatic head and ensured adequate drainage of the obstructed ducts. A Roux-en-Y jejunal limb was prepared by transecting the jejunum approximately 20 cm distal to the ligament of Treitz and brought retrocolically to the pancreatic bed. A longitudinal side-to-side pancreaticojejunal anastomosis was created, extending from the pancreatic tail to the excavated cavity of the head, followed by an end-to-side jejunojejunal anastomosis 20 cm distal to the first anastomosis. A closed-suction drain was placed adjacent to the pancreaticojejunal anastomosis, and drainage amylase levels were monitored postoperatively to determine the timing of drain removal.

### 2.3. Statistical Analysis

Statistical analyses were performed using SPSS version 27.0 and GraphPad Prism version 10.1.2. Quantitative variables were tested for normality. Data conforming to a normal distribution were expressed as mean ± standard deviation (X ± S), and intergroup comparisons were performed using the independent-samples *t*-test. Data not conforming to a normal distribution were expressed as median (first quartile, third quartile) [M (Q1, Q3)], and intergroup comparisons were conducted using the Mann–Whitney *U* test. Categorical variables were presented as frequencies, with intergroup comparisons performed using the Pearson chi-square test; for variables not meeting the assumptions of the Pearson chi-square test, the continuity correction chi-square test was applied. A *p* value < 0.05 was considered statistically significant. Kaplan–Meier survival curves were constructed to compare the intervention-free survival rates between the ERCP and Frey procedure groups, and the differences were analyzed using the log-rank test.

## 3. Results

The analysis (Table 1) revealed a statistically significant difference in the degree of main pancreatic duct dilatation between the ERCP group and the Frey group (*p* < 0.001) and the duration of treatment (*p* < 0.001). No significant differences were observed between the two groups in terms of gender, age, height, weight, BMI, stone type, stone size, stone location, WBC count, CRP, AMY, LIP, ALT, TB, FBG, or ALB.

In terms of postoperative recovery at one week (Table 2), there were no statistically significant differences between the two groups in WBC, CRP, AMY, LIP, ALT, TB, FBG, or ALB, with all parameters returning to normal levels.

In terms of postoperative complications (Table 3), the number of reinterventions was significantly higher in the ERCP group (3.98 ± 0.52) compared with the Frey group (1.27 ± 0.91) (*p* < 0.001). Stone recurrence occurred in 13 patients (40.63%) in the ERCP group and in 3 patients (9.09%) in the Frey group, with a statistically significant difference (*p* = 0.003). There were no statistically significant differences between the ERCP group and the Frey group in terms of postoperative pancreatitis, postoperative bleeding, pancreatic fistula, intra-abdominal infection, or pain relief.

**Figure 2:**
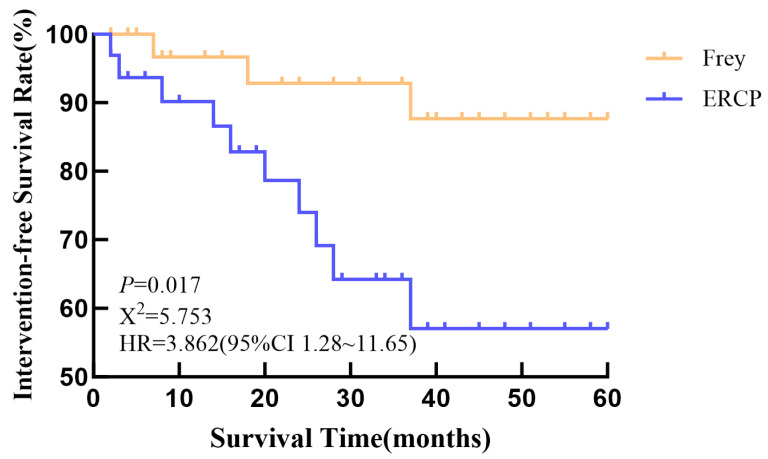
Kaplan–Meier analysis of postoperative intervention-free survival in the ERCP and Frey groups.

In terms of postoperative intervention-free survival, the 1-, 3-, and 5-year intervention-free survival rates in the ERCP group were 90.18%, 64.17%, and 57.04%, respectively. In the Frey group, the corresponding rates were 96.67%, 92.80%, and 87.64%. A statistically significant difference in 5-year intervention-free survival was observed between the two groups (*p* = 0.017) (Figure 2).

## 4. Discussion

The pathogenesis of PDS remains incompletely understood. Current studies suggest that decreased secretion of pancreatic stone protein, increased secretion of lactoferrin in pancreatic juice, pancreatic duct obstruction, and elevated calcium concentration may contribute to stone formation. These factors are generally considered to be associated with alcoholism, CP, malnutrition, biliary tract diseases, and anatomical abnormalities [5,6,7]. In this study, all patients were concomitantly diagnosed with CP. CP is a pathological fibro-inflammatory condition of the pancreas that occurs in individuals with genetic, environmental, and other predisposing risk factors [8]. Irreversible structural changes caused by CP can lead to pain syndromes, characterized by recurrent or persistent pain, and may progress to exocrine and/or endocrine pancreatic insufficiency. Reportedly, genetic mutations and anatomical variations of the pancreatic duct are common risk factors in pediatric patients with CP [9].

ERCP has become the preferred first-line treatment for adult due to its minimally invasive nature, offering stone extraction and ductal decompression with reduced recovery time [10,11]. However, its priority in children is less well-established [12,13,14]. At the same time, whether repeated ERCP procedures and ERCP-based treatments during childhood lead to repeated stimulation of the duodenal major papilla and increase the risk of malignancy in adulthood remains unknown. The Frey procedure is one of the commonly used surgical approaches for treating CP. It is characterized by a duodenum-preserving pancreatic head resection combined with a longitudinal pancreaticojejunostomy involving the body and tail of the pancreas [15]. The objectives of surgery for CP include: alleviating pain, managing pancreatitis-related complications involving adjacent organs, preserving exocrine and endocrine function as much as possible, facilitating social and occupational rehabilitation, and improving overall quality of life [16]. Both ERCP and Frey procedures have demonstrated favorable therapeutic outcomes in the management of CP in adults and are recommended by relevant clinical guidelines [17,18,19,20,21]. However, there is still a lack of consensus regarding the treatment of CP in children [22,23].

In children with chronic pancreatitis, the Frey procedure offers clear advantages over the Puestow procedure or total pancreatectomy with islet autotransplantation (TP IAT) [24,25,26,27,28]. Unlike Puestow, which only drains the pancreatic duct, Frey combines ductal drainage with coring of the pancreatic head, effectively addressing head-dominant disease and ductal stones. This approach provides higher rates of sustained pain relief and lower recurrence, reducing the need for repeat interventions. Frey also preserves pancreatic tissue, minimizing endocrine and exocrine insufficiency. In contrast, TP IAT is more invasive and may require lifelong enzyme or insulin replacement, which is particularly important to avoid in growing children. Additionally, Frey is technically adaptable, with moderate operative time, manageable blood loss, and rapid recovery, making it suitable for pediatric patients. For children with head-dominant disease, ductal stones, and mild-to-moderate ductal dilatation, Frey achieves effective symptom control while preserving function, representing a preferred surgical option.

This study compared the therapeutic outcomes of patients with PDS treated with ERCP or the Frey procedure. The findings indicate that the Frey procedure offers greater advantages in managing patients with more severe main pancreatic duct dilatation [29,30]. Dilatation of the main pancreatic duct is often accompanied by multiple, larger, and deeper stones, in which case ERCP has certain limitations. ERCP primarily alleviates symptoms through stone extraction and stent placement [31], but it does not address the underlying etiology, resulting in a higher likelihood of postoperative recurrence. However, ERCP still offers significant advantages in the management of single and small PDS [32]. The etiology of such PDS is often relatively simple, without complex anatomical variations. In most cases, one to three ERCP procedures can provide long-term benefits for patients, thereby avoiding the considerable trauma associated with open surgery. However, compared with the Frey procedure, ERCP requires a longer treatment period. Regarding postoperative complications, the complication rates in both the ERCP and Frey groups were generally consistent with those reported in the literature [8,12,13,26,33,34,35,36,37]. This indicates that pediatric patients have achieved favorable therapeutic outcomes with either ERCP or Frey procedures for the treatment of PDS. Regarding stone recurrence, the ERCP group had a higher recurrence rate compared with the Frey group. In terms of postoperative interventions, the ERCP group required more interventions than the Frey group. In this study, the number of reinterventions in the ERCP group was higher than that reported in the literature [38], which may be associated with the higher proportion of multiple PDS in our cohort. ERCP removes stones under direct endoscopic visualization; however, for deeply located stones or those in segments that are difficult for the endoscope to access, complete removal is often not achievable in a single procedure. Consequently, multiple interventions are frequently required to achieve sustained therapeutic efficacy.

Regarding postoperative intervention-free survival, the outcomes in the ERCP group were consistent with those of the Frey group and with previously reported results in the literature [16,18,36,39]. This demonstrates the advantages of the Frey procedure in treating complex and multiple PDS. In the surgical management of CP, the Frey procedure is technically simpler than other surgical approaches (such as the Whipple and Beger procedures) and provides comparable outcomes, while carrying lower operative risks and preserving more of the native organ tissue [40,41,42]. Therefore, the Frey procedure is considered a safe and feasible option, capable of achieving better short-term pain control during the perioperative period. In summary, the Frey procedure has become one of the preferred surgical options for the management of complex CP [25].

## 5. Conclusions

Both ERCP and the Frey procedure achieve favorable overall outcomes in the treatment of pediatric PDS. For patients with mild ductal dilatation and first-onset disease, ERCP is preferred, as it offers maximal benefit with minimal invasiveness. However, for patients with marked ductal dilatation or multiple recurrences in whom ERCP has shown suboptimal efficacy, the Frey procedure is recommended to achieve more durable therapeutic results.

## Figures and Tables

**Table 1:** Comparison of Preoperative Clinical Characteristics Between the ERCP and Frey Groups.

Clinical Data	ERCP (n = 32)	Frey (n = 33)	*p*	Test Statistic
Gender (n)			0.914	0.012
Male	18	19		
Female	14	14		
Age (y)	13 (8.77, 16)	14(12, 15.5)	0.425	0.798
Height (cm)	157 (132.25, 163.25)	160 (148.5, 167)	0.215	1.213
Weight (kg)	39.86 ± 15.94	43.70 ± 14.27	0.310	0.694
BMI	17.46 (14.41, 18.75)	17.35 (14.85, 18.95)	0.808	0.243
Stone Type (n)			0.384	0.757
Single	13	10		
Multiple	19	23		
Stone Size (mm)	7.12 ± 1.08	7.67 ± 1.49	0.094	1.699
Stone Location			0.524	1.294
Head	24	22		
Body	8	10		
Tail	0	1		
DMPDD (mm)	6.45 ± 1.24	9.11 ± 1.38	<0.001	8.166
DT (m)	4.4 (2.3, 6.7)	1.8 (0.9, 2.9)	<0.001	4.822
WBC (×10^9^/L)	7.04 (4.92, 8.72)	6.17 (5.24, 8.3)	0.568	−0.571
CRP (mg/L)	7.23 (3.48, 10.97)	7.56 (4.01, 11.35)	0.096	0.052
AMY (U/L)	151 (71, 385)	109 (60, 310)	0.201	−1.280
LIP (U/L)	127 (65, 276)	138 (72, 245)	0.679	0.420
ALT (U/L)	35 (28, 81)	37 (25, 94)	0.660	0.446
TB (μmol/L)	16.8 (12.1, 25.1)	18.1 (11.9, 26.4)	0.332	0.978
FBG (mmol/L)	5.16 (4.19, 6.38)	5.21 (4.35, 6.51)	0.273	1.102
ALB (g/L)	37 (33, 42)	36 (32, 43)	0.372	−0.899

**Table 2:** Comparison of Postoperative Recovery at One Week between ERCP and Frey Groups.

Clinical Data	ERCP (n = 32)	Frey (n = 33)	*p*	Test Statistic
WBC (×10^9^/L)	6.11 (4.28, 7.13)	5.35 (4.36, 7.64)	0.922	−0.105
CRP (mg/L)	1.35 (0.87, 2.02)	1.69 (0.98, 2.25)	0.906	−0.125
AMY (U/L)	52 (31, 78)	49 (32, 69)	0.494	0.689
LIP (U/L)	38 (21, 52)	35 (18, 55)	0.404	0.840
ALT (U/L)	19 (8, 33)	17 (7, 32)	0.896	−0.138
ALT (μmol/L)	10.6 (6.2, 15.3)	11.3 (7.8, 16.4)	0.586	−0.551
FBG (mmol/L)	4.93 (4.28, 5.91)	5.03 (4.41, 5.89)	0.894	0.138
ALB (g/L)	42 (38, 47)	41 (38, 49)	0.579	0.558

**Table 3:** Comparison of Postoperative Outcomes Between ERCP and Frey Groups.

Outcomes	ERCP (n = 32)	Frey (n = 33)	*p*	Test Statistic
PP (n)	3	3	0.968	0.002
PB (n)	1	2	0.573	0.318
PF (n)	0	1	0.321	0.985
IAI (n)	0	2	0.486	0.485
PR (n)	28	30	0.966	0.003
SR (n)	13	3	0.003	8.706
MI (times)	3.98 ± 0.52	1.27 ± 0.91	<0.001	14.68

## Data Availability

The datasets used or analyzed during the study are available from the corresponding author on reasonable request.

## Abbreviations

ERCP	Endoscopic retrograde cholangiopancreatography
PDS	Pancreatic duct stones
CP	Chronic pancreatitis
BMI	Body mass index
DMPDD	Degree of main pancreatic duct dilatation
DT	Duration of treatment
WBC	White blood cell
CRP	C-reactive protein
AMY	Amylase
LIP	Lipase
ALT	Alanine aminotransferase
TB	Total bilirubin
FBG	Fasting blood glucose
ALB	Albumin
PR	Pain relief
MI	Multiple interventions
PP	Postoperative pancreatitis
PB	Postoperative bleeding
PF	Pancreatic fistula
IAI	Intra-abdominal infection
SR	Stone recurrence
TP-IAT	Total pancreatectomy with islet autotransplantation

## References

[1] Beyna T., Neuhaus H., Gerges C. (2018). Endoscopic treatment of pancreatic duct stones under direct vision: Revolution or resignation? Systematic review. Dig. Endosc..

[2] Tandan M., Nageshwar Reddy D. (2013). Endotherapy in chronic pancreatitis. World J. Gastroenterol..

[3] Ammann R.W., Muench R., Otto R., Buehler H., Freiburghaus A.U., Siegenthaler W. (1988). Evolution and regression of pancreatic calcification in chronic pancreatitis. A prospective long-term study of 107 patients. Gastroenterology.

[4] Rösch T., Daniel S., Scholz M., Huibregtse K., Smits M., Schneider T., Ell C., Haber G., Riemann J.F., Jakobs R. (2002). Endoscopic treatment of chronic pancreatitis: A multicenter study of 1000 patients with long-term follow-up. Endoscopy.

[5] Makita S., Amano H., Kawashima H., Hinoki A., Shirota C., Tainaka T., Sumida W., Yokota K., Okamoto M., Takimoto A. (2022). Utility of endoscopic retrograde cholangiopancreatography in management of pediatric pancreaticobiliary disease. BMC Pediatr..

[6] Boam T., Gabriel M., Rogoyski B.G., Ram A.D., Awan A. (2022). Surgical drainage procedures for paediatric chronic pancreatitis: A scoping review. Pediatr. Surg. Int..

[7] Ceppa E.P., Pitt H.A., Hunter J.L., Leys C.M., Zyromski N.J., Rescorla F.J., Sandrasegaran K., Fogel E.L., McHenry L.W., Watkins J.L. (2013). Hereditary pancreatitis: Endoscopic and surgical management. J. Gastrointest. Surg..

[8] Kohoutova D., Tringali A., Papparella G., Perri V., Boškoski I., Hamanaka J., Costamagna G. (2019). Endoscopic treatment of chronic pancreatitis in pediatric population: Long-term efficacy and safety. United Eur. Gastroenterol. J..

[9] Randall M.M., McDaniels S., Kyle K., Michael M., Giacopuzzi J., Brown L.A. (2019). Pancreatitis in pre-adolescent children: A 10 year experience in the pediatric emergency department. BMC Emerg. Med..

[10] Buxbaum J.L., Freeman M., Amateau S.K., Chalhoub J.M., Coelho-Prabhu N., Desai M., Elhanafi S.E., Forbes N., Fujii-Lau L.L., Kohli D.R. (2023). American Society for Gastrointestinal Endoscopy guideline on post-ERCP pancreatitis prevention strategies: Summary and recommendations. Gastrointest. Endosc..

[11] Sheth S.G., Machicado J.D., Chalhoub J.M., Forsmark C., Zyromski N., Thosani N.C., Thiruvengadam N.R., Ruan W., Pawa S., Ngamruengphong S. (2024). American Society for Gastrointestinal Endoscopy guideline on the role of endoscopy in the management of chronic pancreatitis: Summary and recommendations. Gastrointest. Endosc..

[12] Hosseini A., Sohouli M.H., Sharifi E., Sayyari A., Sridharan K., Tajalli S., Imanzadeh N., Fatahi S. (2023). Indications, success, and adverse event rates of pediatric endoscopic retrograde cholangiopancreatography (ERCP): A systematic review and meta-analysis. BMC Pediatr..

[13] Keane M.G., Kumar M., Cieplik N., Thorburn D., Johnson G.J., Webster G.J., Chapman M.H., Lindley K.J., Pereira S.P. (2018). Paediatric pancreaticobiliary endoscopy: A 21-year experience from a tertiary hepatobiliary centre and systematic literature review. BMC Pediatr..

[14] Rosen J.D., Lane R.S., Martinez J.M., Perez E.A., Tashiro J., Wagenaar A.E., Van Haren R.M., Kumar A., Sola J.E. (2017). Success and safety of endoscopic retrograde cholangiopancreatography in children. J. Pediatr. Surg..

[15] Frey C.F., Smith G.J. (1987). Description and rationale of a new operation for chronic pancreatitis. Pancreas.

[16] Ray S., Ansari Z., Kumar D., Jana K., Khamrui S. (2020). Short- and long-term outcome of surgery for chronic pancreatitis in children: A single surgeon experience. Pediatr. Surg. Int..

[17] Tan C.-L., Zhang H., Li K.-Z. (2015). Single center experience in selecting the laparoscopic Frey procedure for chronic pancreatitis. World J. Gastroenterol..

[18] Kempeneers M.A., van Hemert A.K.E., van der Hoek M., Issa Y., van Hooft J.E., Nio C.Y., Busch O.R., van Santvoort H.C., Besselink M.G., Boermeester M.A. (2022). Short- and long-term outcomes of selective use of Frey or extended lateral pancreaticojejunostomy in chronic pancreatitis. Br. J. Surg..

[19] Li Q., Li S., Hou S., Zhang L., Chen S., Wang J., Lv J., Wu Y., Huang Q., Li Y. (2024). ERCP-Related adverse events in pediatric patients: A 10-years single-site review. Pediatr. Surg. Int..

[20] Poddar U., Thapa B.R., Bhasin D.K., Prasad A., Nagi B., Singh K. (2001). Endoscopic retrograde cholangiopancreatography in the management of pancreaticobiliary disorders in children. J. Gastroenterol. Hepatol..

[21] Kempeneers M.A., Issa Y., Ali U.A., Baron R.D., Besselink M.G., Büchler M., Erkan M., Fernandez-Del Castillo C., Isaji S., Izbicki J. (2020). International consensus guidelines for surgery and the timing of intervention in chronic pancreatitis. Pancreatology.

[22] Durakbasa C.U., Balik E., Yamaner S., Bulut T., Büyükuncu Y., Sökücü N., Akyüz A., Bugra D. (2008). Diagnostic and therapeutic endoscopic retrograde cholangiopancreatography (ERCP) in children and adolescents: Experience in a single institution. Eur. J. Pediatr. Surg..

[23] Rocca R., Castellino F., Daperno M., Masoero G., Sostegni R., Ercole E., Lavagna A., Barbera C., Canavese F., Pera A. (2005). Therapeutic ERCP in paediatric patients. Dig. Liver Dis..

[24] Rollins M.D., Meyers R.L. (2004). Frey procedure for surgical management of chronic pancreatitis in children. J. Pediatr. Surg..

[25] Ray S., Sanyal S., Ghatak S., Khamrui S., Biswas J., Saha S., Mandal T.S., Chattopadhyay G. (2015). Frey procedure for chronic pancreatitis in children: A single center experience. J. Pediatr. Surg..

[26] Zhou Y., Shi B., Wu L., Wu X., Li Y. (2015). Frey procedure for chronic pancreatitis: Evidence-based assessment of short- and long-term results in comparison to pancreatoduodenectomy and Beger procedure: A meta-analysis. Pancreatology.

[27] Jeropoulos R.M., Joshi D., Aldeiri B., Davenport M. (2024). Surgical and Endoscopic Intervention for Chronic Pancreatitis in Children: The Kings College Hospital Experience. Children.

[28] Pappas S.G., Pilgrim C.H.C., Keim R., Harris R., Wilson S., Turaga K., Tsai S., Dua K., Khan A., Oh Y. (2013). The Frey procedure for chronic pancreatitis secondary to pancreas divisum. JAMA Surg..

[29] Ray S., Basu C., Dhali A., Dhali G.K. (2022). Frey procedure for chronic pancreatitis: A narrative review. Ann. Med. Surg..

[30] Chen G., You Y., Yan H., He J., Gong J., Wei S. (2020). Drainage procedure for pancreatolithiasis: Re-examination of the pancreatic duct diameter standard. Ann. Surg. Treat. Res..

[31] Green J.A., Scheeres D.E., Conrad H.A., Cloney D.L., Schlatter M.G. (2007). Pediatric ERCP in a multidisciplinary community setting: Experience with a fellowship-trained general surgeon. Surg. Endosc..

[32] Rieder S., Michalski C.W., Friess H. (2010). Indications for endoscopic or surgical treatment of chronic pancreatitis. Dig. Dis..

[33] Teng R., Yokohata K., Utsunomiya N., Takahata S., Nabae T., Tanaka M. (2000). Endoscopic retrograde cholangiopancreatography in infants and children. J. Gastroenterol..

[34] Iqbal C.W., Baron T.H., Moir C.R., Ishitani M.B. (2009). Post-ERCP pancreatitis in pediatric patients. J. Pediatr. Gastroenterol. Nutr..

[35] Lorio E., Moreau C., Hernandez B., Rabbani T., Michaud K., Hachem J., Aggarwal P., Stolow E., Brown L., Michalek J.E. (2023). Pediatric ERCP: Factors for Success and Complication-A 17-Year, Multisite Experience. J. Pediatr. Gastroenterol. Nutr..

[36] Ueda J., Miyasaka Y., Ohtsuka T., Takahata S., Tanaka M. (2015). Short- and long-term results of the Frey procedure for chronic pancreatitis. J. Hepatobiliary Pancreat. Sci..

[37] Khatkov I., Izrailov R., Tsvirkun V., Alikhanov R., Vasnev O., Dyuzheva T., Egorov V., Dalgatov K., Baychorov M., Agami P. (2022). Laparoscopic versus open Frey procedure: Comparative analysis of short and long-term outcomes. Pancreatology.

[38] Guo H., Luo J., Yang H., Yang J., Bian H., Duan X., Wang X. (2025). Pediatric endoscopic retrograde pancreatography expertise in chronic pancreatitis: A single-center analysis. Front. Pediatr..

[39] Suzumura K., Hatano E., Okada T., Asano Y., Uyama N., Nakamura I., Hai S., Fujimoto J. (2018). Short- and long-term outcomes of the Frey procedure for chronic pancreatitis: A single-center experience and summary of outcomes in Japan. Surg. Today.

[40] Diener M.K., Rahbari N.N., Fischer L., Antes G., Büchler M.W., Seiler C.M. (2008). Duodenum-preserving pancreatic head resection versus pancreatoduodenectomy for surgical treatment of chronic pancreatitis: A systematic review and meta-analysis. Ann. Surg..

[41] Allen C.J., Yakoub D., Macedo F.I., Dosch A.R., Brosch J., Dudeja V., Ayala R., Merchant N.B. (2018). Long-term Quality of Life and Gastrointestinal Functional Outcomes After Pancreaticoduodenectomy. Ann. Surg..

[42] Strate T., Bachmann K., Busch P., Mann O., Schneider C., Bruhn J.P., Yekebas E., Kuechler T., Bloechle C., Izbicki J.R. (2008). Resection vs drainage in treatment of chronic pancreatitis: Long-term results of a randomized trial. Gastroenterology.

